# Preliminary evaluation of urinary soluble Met as a Biomarker for urothelial carcinoma of the bladder

**DOI:** 10.1186/1479-5876-12-199

**Published:** 2014-10-21

**Authors:** Brian K McNeil, Maximiliano Sorbellini, Robert L Grubb, Andrea Apolo, Fabiola Cecchi, Gagani Athauda, Benjamin Cohen, Alessio Giubellino, Haley Simpson, Piyush K Agarwal, Jonathan Coleman, Robert H Getzenberg, George J Netto, Joanna Shih, W Marston Linehan, Peter A Pinto, Donald P Bottaro

**Affiliations:** Urologic Oncology Branch, Center for Cancer Research, National Cancer Institute, Bldg 10, Hatfield Clinical Research Center, Rm 2 W-3952 10 Center Drive MSC 1210, 20892-1210 Bethesda, MD USA; Division of Urology, Washington University, St. Louis, MO USA; Medical Oncology Branch, Center for Cancer Research, National Cancer Institute, Bethesda, MD USA; Brady Urological Institute, The Johns Hopkins University School of Medicine, Baltimore, MD USA; Department of Pathology, The Johns Hopkins University School of Medicine, Baltimore, MD USA; Biometric Research Branch, Division of Cancer Diagnosis and Treatment, National Cancer Institute, Rockville, MD USA

**Keywords:** Urothelial carcinoma, Bladder cancer, Biomarker, HGF receptor, Met, Urine

## Abstract

**Background:**

Among genitourinary malignancies, bladder cancer (BCa) ranks second in both prevalence and cause of death. Biomarkers of BCa for diagnosis, prognosis and disease surveillance could potentially help prevent progression, improve survival rates and reduce health care costs. Among several oncogenic signaling pathways implicated in BCa progression is that of hepatocyte growth factor (HGF) and its cell surface receptor, Met, now targeted by 25 experimental anti-cancer agents in human clinical trials. The involvement of this pathway in several cancers is likely to preclude the use of urinary soluble Met (sMet), which has been correlated with malignancy, for initial BCa screening. However, its potential utility as an aid to disease surveillance and to identify patients likely to benefit from HGF/Met-targeted therapies provide the rationale for this preliminary retrospective study comparing sMet levels between benign conditions and primary BCa, and in BCa cases, between different disease stages.

**Methods:**

Normally voided urine samples were collected from patients with BCa (Total: 183; pTa: 55, pTis: 62, pT1: 24, pT2: 42) and without BCa (Total: 83) on tissue-procurement protocols at three institutions and sMet was measured and normalized to urinary creatinine. Normalized sMet values grouped by pathologic stage were compared using non-parametric tests for correlation and significant difference. ROC analyses were used to derive classification models for patients with or without BCa and patients with or without muscle-invasive BCa (MIBCa or NMIBCa).

**Results:**

Urinary sMet levels accurately distinguished patients with BCa from those without (p < 0.0001, area under the curve (AUC): 0.7008) with limited sensitivity (61%) and moderate specificity (76%), and patients with MIBCa (n = 42) from those with NMIBCa (n = 141; p < 0.0001, AUC: 0.8002) with moderate sensitivity and specificity (76% and 77%, respectively) and low false negative rate (8%).

**Conclusions:**

Urinary sMet levels distinguish patients with BCa from those without, and patients with or without MIBCa, suggesting the potential utility of urinary sMet as a BCa biomarker for surveillance following initial treatment. Further studies are warranted to determine its potential value for prognosis in advanced disease, predicting treatment response, or identifying patients likely to benefit from Met-targeted therapies.

## Background

In 2013, 72,570 new cases of bladder cancer (BCa) and 15,210 bladder cancer-related deaths were estimated in the U.S. alone [1]. In approximately 70% of newly diagnosed bladder cancer cases, disease is confined to the mucosa, but requires long term monitoring by cystoscopy to detect frequent recurrences. The remaining 30% of new cases present at a more advanced stage, with muscle-invasion, locoregional nodal involvement or distant metastases. About half of those individuals with muscle-invasive bladder cancer fail surgical or chemoradiation definitive therapy within 5 years and succumb to the disease [1, 2]. The 5- and 10-year survival rates for patients with lymph node involvement are 31 and 23%, respectively [2]. Combination platinum-based chemotherapy, the standard or care for patients with metastatic disease, provides a median survival of only 15 months and a 5-year survival rate of less than 15% [3]. BCa has the highest costs per patient in the U.S. compared to all other cancers, reflecting disease prevalence as well as costs of long term monitoring [4]. These circumstances underscore the urgent need for finding novel disease biomarkers and treatments.

The cell surface receptor tyrosine kinase for hepatocyte growth factor (HGF), known as Met, is widely present in cells of epithelial origin. HGF/Met signaling is required for normal development and adult homeostasis but is also frequently implicated in cancer, contributing to tumor invasiveness and metastasis [5]. Evidence of HGF/Met pathway involvement in BCa has been found in model systems [6–8] and *in vivo* [9–11]. In a prior study we found that proteolytic shedding of soluble Met (sMet) ectodomain from cells increased with transformation and correlated with malignancy, and we developed a high-throughput, two-site immunoassay for Met ectodomain detection with four-log linearity and attomole sensitivity [12]. This assay is ideally suited to low protein samples such as urine, where, in constant contact with the urothelium, even small changes in sMet might be detected. The goal of the retrospective study described here was to obtain an initial assessment of urinary sMet level as a potential biomarker of urothelial carcinoma of the bladder using normally voided, tissue banked specimens collected at urology departments of three institutions. The findings that urinary sMet levels were significantly higher in BCa patients than in individuals with no evidence of cancer, higher in muscle-invasive than in non-invasive cancer, and higher in more malignant pathologic stages, suggest that follow-up prospective clinical studies to investigate its potential utility in disease prognosis, as an aid to disease surveillance, and to identify patients likely to benefit from HGF/Met-targeted therapies, are warranted.

## Methods

### Patients and Samples

From 2005 to 2012, normally voided urine samples were collected from patients enrolled in IRB approved tissue procurement protocols at the National Cancer Institute, the Brady Urological Institute of The Johns Hopkins University School of Medicine and Washington University in St. Louis, by informed consent prior to cystoscopy, transurethral resection or cystectomy. sMet values were normalized to urinary creatinine values obtained by standard clinical tests on the same sample. Only patients with a pathology confirmation of UC of the bladder were included in the final analysis; reports were reviewed as they appeared in electronic medical records. Patients with other known cancers in addition to bladder cancer were excluded. The control group was composed of patients with benign bladder pathology and not known to have any other malignancy.

### Two-site electrochemiluminescent sMet immunoassay

Normally voided urine samples were stored at −80°C prior to analysis. Thawed samples were processed by centrifugation, ultrafiltration and pH adjustment to 7.0 prior to sMet quantitation as described previously [12]. Briefly, streptavidin-coated 96-well plates were blocked, washed with PBS and coated with a biotin-tagged, affinity-purified human Met ectodomain-specific capture antibody (BAF358, R&D Systems). Wells were washed again before adding sample or sMet standard (358-MT, R&D Systems) for 1 h with shaking. After washing with PBS, detection antibody (AF276, R&D Systems) labeled with MSD-Sulfo-Tag (Meso Scale Discovery) was added for 1 h with shaking. Wells were washed with PBS before adding read buffer and plates were read in a MSD Sector Imager 2400. Assays were performed blinded to study endpoint.

### Statistical analysis

**Figure 1:**
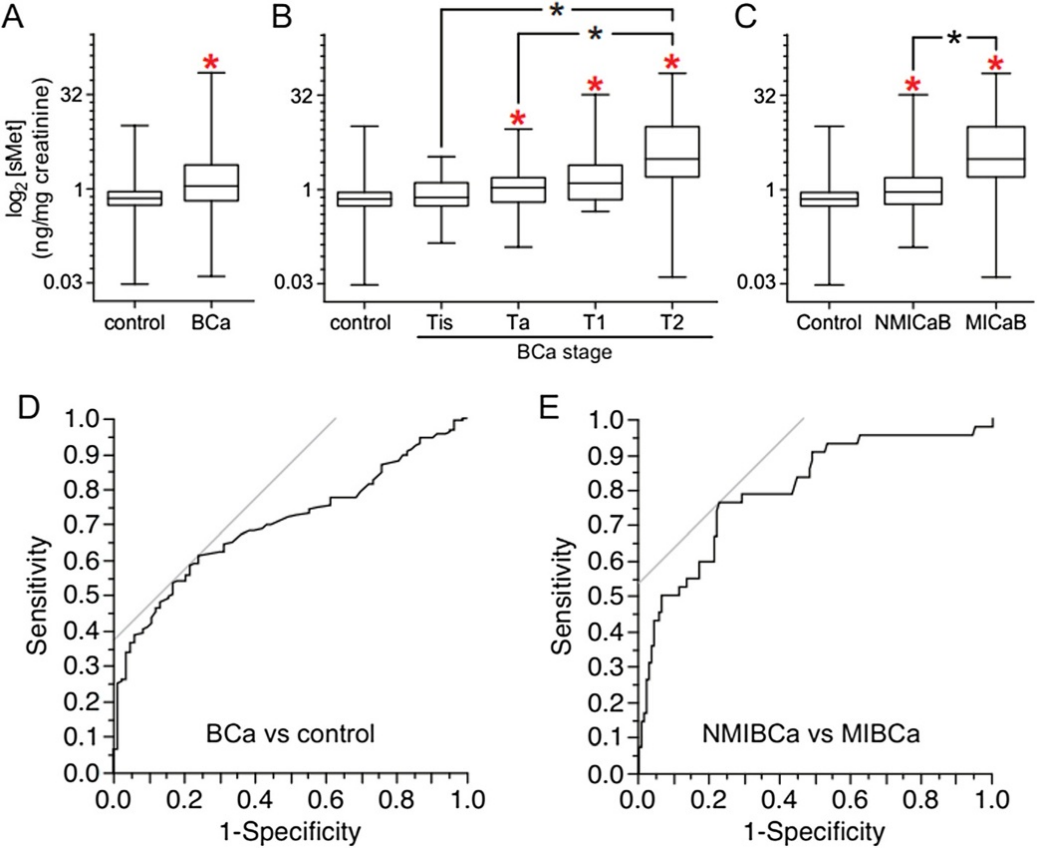
**Comparison of urinary sMet levels among BCa patients and controls. A**. Box and whisker plot of urine sMet/creatinine values (ng sMet/mg creatinine) for control patients with no evidence of BCa (n = 83) and patients with pathology proven BCa (n = 183). Red asterisk indicates significant difference in median value from control (Mann–Whitney test with Bonferroni multiple test correction). **B**. Box and whisker plots of urine sMet/creatinine values (ng sMet/mg creatinine) for control patients with no evidence of BCa and patients with BCa subdivided by pathologic stage. N for each group are listed in Table 1. Red asterisks indicate significant difference in median value from control; black asterisks indicate significant differences among medians as indicated by brackets (Mann–Whitney test with Bonferroni multiple test correction). **C**. Box and whisker plot of urine sMet/creatinine values (ng sMet/mg creatinine) for control patients with no evidence of BCa (n = 83), patients with NMIBCa (n = 141) or with MIBCa (n = 42). Red asterisks indicate significant difference in median value from control; black asterisk indicates significant differences among medians as indicated by brackets (Mann–Whitney test with Bonferroni multiple test correction). **D**. ROC curve of urine sMet/creatinine values (ng sMet/mg creatinine) for control patients with no evidence of BCa (n = 83) and patients with pathology proven BCa (n = 183). **E**. ROC curve of urine sMet/creatinine values (ng sMet/mg creatinine) for patients with NMIBCa (n = 141) or with MIBCa (n = 42). Criterion values balancing sensitivity and specificity are indicated by the gray lines tangent to the ROC curves.

Statistical analyses were performed using JMP (SAS Institute Inc.), Excel (Microsoft Inc.) and Prism (GraphPad Software Inc.). Wilcoxon, Mann Whitney and Kruskal-Wallis tests, in conjunction with the Bonferroni multiple test correction, were used to determine statistical differences between groups. Assuming a single test significance level of 0.05, the Bonferrroni multiple test correction for 13 combined tests (10 as shown in Table 1 plus control vs. BCa and NMIBCa vs. MIBCa, and control vs tumor grade, high or low) gives an adjusted significance level of 0.0038; this value was used in denoting significant differences in the text and Figure 1A,B and C. Receiver Operating Curves (ROC) were calculated and Area Under the Curve (AUC) determined using JMP. Cutoff values determined using a 45° line drawn tangential to the ROC curve (Figure 1D and E) were used to develop specificity, sensitivity and prediction values.

**Table 1:** **Mann–Whitney p values for difference comparisons in urinary median sMet/creatinine levels among controls and BCa stages**

	Control (n = 83)	Tis (n = 62)	Ta (n = 55)	T1 (n = 24)
**Tis**	0.2213			
**Ta**	0.0002	0.0367		
**T1**	0.0003	0.0110	0.2474	
**T2 (n = 42)**	<0.0001	<0.0001	<0.0001	0.0127

## Results

Urine samples for sMet analysis were obtained from 183 patients with BCa (pTa: 55, pTis: 62, pT1: 24, pT2: 42) and 83 patients without evidence of bladder cancer (control). Median urine sMet/creatinine values (ng/mg) were significantly different between control and all BCa patients (Figure 1A; p < 0.0001) and between controls and BCa patients with disease stage of Ta, T1 or T2 (Figure 1B). Mann–Whitney p values for differences among controls and each pathologic stage are listed in Table 1. With the exception of Tis, the results indicate progressively increasing sMet/creatinine values between stages. Although samples were obtained from patients with stage T3 or T4a disease, many of these patients had undergone radical cystectomy, and differences in sMet levels observed between these and normally voided samples suggested that an independent study to distinguish the effects of cystectomy from those of BCa on sMet level was needed; therefore only patients with intact bladders and BCa stage T2 or lower were included in this study. BCG treatment was also observed to transiently elevate urinary sMet levels; only samples from patients that had received BCG therapy more than one month prior were included in the final analysis.

Consistent with the known role of HGF/Met signaling in tumor invasiveness and the correlation between Met shedding and malignancy found previously, the median urinary sMet/creatinine value of patients with non-muscle invasive BCa (NMIBCa; n = 141) was significantly lower than that of patients with muscle-invasive BCa (MIBCa; n = 42; p < 0.0001; Figure 1C). Both BCa groups were significantly higher than control (control vs NMIBCa p = 0.0004; control vs MIBCa p < 0.0001; Figure 1C). Significant differences in median urinary sMet levels were also found between control (n = 83) and low grade tumor groups (n = 25; p = 0.0001) and between control and high grade tumor groups (n = 59; p = 0.0013), but not between low grade and high grade tumor groups. Comparison of median sMet levels in subjects with no cytologic atypia to those with any degree of atypia (atypia, suspicious for cancer or positive for cancer) showed a trend towards statistical significance (p = 0.0087) but did not meet the significance threshold adjustment for 14 correlative tests (p < 0.0036); these results were further limited as only 48/183 (26.2%) of subjects had cytologic analysis performed.

ROC analysis of control vs BCa groups (Figure 1D) yielded an AUC of 0.7008. A criterion value of > 0.920 to classify patients as benign or having BCa yielded 61.2% sensitivity and 75.9% specificity (Table 2). Similar analysis of NMIBCa vs MIBCa groups (Figure 1E) yielded an AUC of 0.8002, and a criterion value of > 1.650 yielded 76.2% sensitivity and 77.3% specificity (Table 2).

**Table 2:** **Sensitivity, specificity, positive (PPV) and negative predictive values (NPV), and Area Under the Curves (AUCs) derived from ROC analyses of sMet/creatinine value comparisons**

Comparison	Criterion	Sensitivity	Specificity	PPV	NPV	AUC
Control vs BCa	> 0.920	0.6120	0.7590	0.8485	0.4701	0.7008
NMIBCa vs MIBCa	> 1.650	0.7619	0.7730	0.5000	0.9160	0.8002

## Discussion

There are currently over 500,000 cases of bladder cancer in the U.S., and this disease also has the highest cost per patient across all types of cancer [4] due to in large part to continuous invasive cystoscopic surveillance of non-muscle invasive disease. Non muscle-invasive tumors have a high rate of recurrence (up to 70%) and more than 15% progress to higher stages, usually muscle-invasive disease. The American Urological Association recommends cystoscopic tumor surveillance every 3–6 months for 3 years and at least yearly thereafter [13], and the US National Comprehensive Cancer Network has made similar recommendations [14]. Surveillance with cystoscopy, and transurethral resection of a bladder lesion (TURBT) when necessary, carries risk of infection and trauma to the lower urinary tract, and these procedures also represent the most dollars allocated for the treatment of bladder cancer [15, 16]. Furthermore, a substantial fraction of patients are managed with an intensity of follow-up and treatment that does not correlate with improved cancer-specific survival or avoidance of a major intervention; in fact, patients who received more intensive treatment were more likely to undergo subsequent radical cystectomy [17, 18]. Thus, a low cost, non-invasive method of bladder cancer detection and surveillance that could diminish the number of procedures being performed would significantly benefit patients and health care systems.

Cytology is currently the most widely used test for the detection of BCa [19]. While cytology is very specific (96%), its usefulness is limited by low sensitivity (44%), particularly for low-grade disease [20]. The interpretation of cytological specimens varies depending on how the specimens were acquired (i.e. via voiding, cystoscopy, ureteral brushings or urethral catheterization), and requires a highly trained pathologist, making the test completely operator dependent; inter-observer agreement has been reported to be highly variable [21]. All current U.S. FDA-approved non-invasive tests for the detection of BCa have higher sensitivities than cytology (FISH: 76%, NMP22: 68%, ImmunoCyt: 84%), but lower specificity (FISH: 85%, NMP22: 79%, ImmunoCyt: 75%) [20, 22]. The estimated sensitivity of the sMet assay for detection of BCa (61%) is lower than the FISH, NMP22 or ImmunoCyt tests. In addition to this limitation, the widespread involvement of HGF/Met signaling in human cancers, particularly those with direct exposure to the urinary tract such as renal cell carcinoma and prostate cancer, raises the likely possibility that other malignancies may be associated with increased urinary sMet [12]. Thus, we do not foresee urinary sMet measurement alone fulfilling the role of an initial BCa screen. It is noteworthy that the multiwell electrochemiluminescent platform used here is well suited to multiplexing sMet measurement with other tests (up to 6 at present) performed simultaneously on the same sample. A multiplexed urine test that met criteria for reliable BCa screening would complement existing diagnostic methods and is widely sought.

More immediately, several attributes indicate the suitability of urinary sMet measurement for BCa surveillance: a very high degree of precision; good specificity (76%); low false positive rate (PPV 85%); good sensitivity to MIBCa (76%) and a very low false negative rate (NPV 92%). Accuracy is based on external reference standards (for sMet and creatinine) that enable longitudinal comparisons between individuals or groups. A reliable biomarker for surveillance would compliment cystoscopy and TURBT and could potentially reduce the frequency of these invasive procedures and their associated costs [23]. The sMet immunoassay is robust, fast, high throughput, and easily automated; the combined materials and labor cost of the two-site immunoassay used in our study was less than $30 per sample. These features, together with the use of commercially available reagents and an established assay platform, indicate simple, low cost implementation by existing clinical laboratories.

The development of prognostic biomarkers and predictive biomarkers for identifying patients most likely to benefit from targeted agents are also active areas of research in urothelial carcinoma. Met abundance has been associated with a worse prognosis for many tumor types and may be associated with more aggressive urothelial tumors [24]; in a limited survey of human urothelial cancer-derived cell lines, we found that Met content correlated directly with disease grade and metastatic potential in mice [25]. The potential prognostic value of urinary sMet as a surrogate of tumor Met expression in BCa patients with an intact bladder is currently under investigation.

Finally, significant correlations between tumor Met content and treatment response have been reported for HGF/Met pathway inhibitors in advanced gastric and lung cancers [5, 26–28]. Although these studies suggest that tumor Met levels may predict response to treatment, they also bring to light potential obstacles to obtaining this information for a large patient population, e.g. limited availability of primary tumor samples, and safety and/or feasibility issues in advanced disease where metastases may be inaccessible for biopsy. We are currently investigating the clinical value of urinary sMet as a prognostic and predictive biomarker in patients with advanced urothelial cancer undergoing Met targeted therapy [26]; NCT01688999.

## Conclusions

Urinary sMet levels distinguish patients with BCa from those without, and patients with or without MIBCa. Because elevated urinary sMet may be associated with malignancies other than BCa, sMet measurement alone is not appropriate as a primary BCa screen. However, urinary sMet may have utility as a BCa biomarker for surveillance following initial diagnosis and treatment. Further clinical studies are warranted to determine the potential utility of urinary sMet for prognosis in more advanced disease, predicting treatment response, and for identifying patients likely to benefit from Met-targeted therapies.
